# Origin of Orthogonality of Strain-Promoted Click Reactions

**DOI:** 10.1002/chem.201501727

**Published:** 2015-07-14

**Authors:** Johannes A Wagner, Davide Mercadante, Ivana Nikić, Edward A Lemke, Frauke Gräter

**Affiliations:** [a] Heidelberg Institute for Theoretical Studies 69118 Heidelberg (Germany), Fax: (+49) 6221-533-298 E-mail: frauke.graeter@h-its.org; [b] Institute for Theoretical Physics, Heidelberg University 69120 Heidelberg (Germany); [c] Interdisciplinary Center for Scientific Computing 69120 Heidelberg (Germany); [d] European Molecular Biology Laboratory 69117 Heidelberg (Germany)

**Keywords:** cycloaddition, inverse electron demand, Diels–Alder reaction, density functional calculations, orbital gap

## Abstract

Site-specific labeling of biomolecules is rapidly advancing due to the discovery of novel mutually orthogonal reactions. Quantum chemistry studies have also increased our understanding of their relative rates, although these have until now been based on highly simplified reactants. Here we examine a set of strain-promoted click-type cycloaddition reactions of *n*-propyl azide, 3-benzyl tetrazine and 3-benzyl-6-methyl tetrazine with cyclooctenes/ynes, in which we aim to address all relevant structural details of the reactants. Our calculations have included the obligatory handles used to attach the label and biomolecule as these can critically influence the stereochemistry and electron demand of the reaction. We systematically computed orbital gaps, activation and distortion energies using density functional theory and determined experimental rates for validation. Our results challenge the current paradigm of the inverse electron demand for this class of reactions. We found that the ubiquitous handles, when next to the triple bond of cyclooctynes, can switch the Diels–Alder type ligations to normal electron demand, a class we term as SPINEDAC reactions. Electron donating substituents on tetrazine can enhance normal demand but also increase distortion penalties. The presence and isomeric configuration of handles thus determine the reaction speed and regioselectivity. Our findings can be directly utilized in engineering genuine cycloaddition click chemistries for biological labeling.

## Introduction

**Figure 1:**
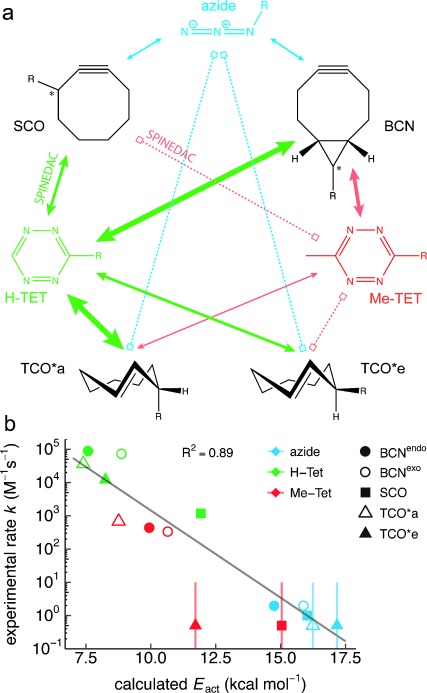
Kinetics of mutually orthogonal click reactions. a) Overview of the investigated reaction set between TCO*e/a isomers, BCN^*endo*/*exo*^ isomers and SCO with azide, H-Tet and Me-Tet. Different isomers are marked as *. Differently sized arrows represent the reaction rates, where thicker means faster. Dashed lines show reactions that were too slow to be measured. Structures of all isomers are given in Figure S1. b) Correlation between experimental rate constants *k* and the calculated activation energies *E*act for each pair of reactants. The solid line shows a linear regression. The SCO/BCN-azide rates are taken from Borrmann et al.[13] For the reactions too slow to be measured, we only obtained an upper limit for *k* as indicated by the error lines and omitted such points for the linear fit. Experimental error bars are smaller than the symbol size and are omitted for clarity.

Cycloadditions are an important class of reactions for site-specific labeling with applications in super-resolution microscopy of cellular components.[1–5] Key properties of these reactions are their bioorthogonality and high selectivity. By exploiting the concept of mutual orthogonality between several click-type cycloaddition reactions, a combination of particularly slow and fast reactions enables the simultaneous labeling of multiple sites in a kinetically controlled fashion in vivo and in vitro.[6–8] Recently, a set of orthogonal reactions has been used by Nikić et al.[9] to label the insulin receptor and Influenza proteins on non-canonical amino acids bearing strained 8-membered rings in a time dependent manner. The click reactions utilized in this work are *n*-propyl azide, 3-benzyl tetrazine (H-Tet) and 3-benzyl-6-methyl tetrazine (Me-Tet) ligations to three types of eight-membered cyclic rings (Figure 1 a and Supporting Information Figure S1, IUPAC names in Supporting Information), namely i) racemic equatorial trans-cyclooct-2-en-1-methylcarbamate (TCO*e) and racemic axial trans-cyclooct-2-en-1-methylcarbamate (TCO*a), ii) two enantiomers of strained cyclooct-2-yn-1-methylcarbamate (SCO), and iii) *endo*- and *exo*-bicyclonon-5-yn-1-methylcarbamate (BCN^*endo*^ & BCN^*exo*^). The tetrazine ligation, a 4+2-cycloaddition, is a strain-promoted inverse electron demand Diels–Alder reaction (SPIEDAC),[10–11] while the alkyne–azide ligation is a strain-promoted Huisgen-type 1,3-dipolar 3+2-cycloaddition (SPAAC).[12–15] The large differences in reaction speed when employing supposedly similar ligation partners, such as H-Tet versus Me-Tet, or TCO* versus SCO, render dual color labeling of biological systems possible.[14, 16]1

Quantitatively predicting such differences and understanding their origin by quantum chemical calculations would increase the ability to further enhance the mutual orthogonality and augment the available set of reactions for preferential labeling. A large body of previous quantum-chemical computational work has characterized and thereby significantly advanced our understanding of a number of highly related strain-promoted click reactions.[17–21] They mostly ascribed the differences in reactivity of these reactions, which have been shown to feature an inverse Diels–Alder electron demand, to contributions from orbital interactions, distortion and Pauli repulsion.[22–23] The higher reactivity of tetrazines towards *trans*-cyclooctene was attributed to their higher electrophilicity as compared to azides,[19] while introducing nitrogens into the diene was proposed to decrease Pauli repulsion, rendering tetrazines the fastest reaction partner for cycloadditions with alkenes.[23]

Notwithstanding these advances, previous work has been focusing on highly simplified reactant scaffolds limiting the comparison of calculated activation energies to reaction rates determined experimentally for more complex scaffolds. However, attaching handles to the functional groups of azides, tetrazines and the different eight-membered rings is the crucial requisite to label biomolecules, and may also critically affect the reactivity of the molecule, as experimental data increasingly certify that the speed of these reactions is highly susceptible to minor changes in substituents.[9] Importantly, handles on cyclooctyne rings introduce a single stereocenter whereas handles on cyclooctene rings add an additional stereocenter. This results in two SCO enantiomers and two pairs of TCO* enantiomers (see Figure S1). The configuration of the stereocenters emerge as a crucial determinant for this class of click reactions.[24–25] Considering the effect of the genuine handles used for functionalization is therefore indispensable for a direct validation of insights from quantum chemical calculations and, more importantly, for the application of these findings to the design of novel orthogonal reactions.

We addressed this fundamental challenge and herein present the first comprehensive set of measured rates and computed barriers for reactants with direct relevance for biological applications. We systematically computed the conformations and energies for the SPIEDAC and SPAAC reactions described above, using density functional theory (DFT). This resulted in a total of 48 reactions with configurationally distinct transition states and products, out of which 24 are chemically distinct enantiomers. The resulting system sizes required advanced sampling of the complex energy landscape, and the diminishing barriers of the high speed TCO* reactions rendered it necessary to incorporate van der Waals complexes into the reaction pathway. On this basis, unexpectedly, we find that SPIEDAC reactions involving SCO and tetrazines to follow normal electron demand, ascribed to the electrophilicity of the SCO. We thus term this class of reactions SPINEDAC (strain-promoted inherently normal electron demand Diels–Alder cycloaddition). We observe isomeric configurations to critically fine-tune the reactivity of the investigated click reactions. Our study aids the engineering of a currently widely used set of cycloadditions to further enhance their reactivity and mutual orthogonality for their applicability in biology.

### Methods

We used the M06-2X density functional[26–27] with the 6-311+G(d,p) basis set[28] and PCM solvent for the quantum chemical calculations carried out in Gaussian 09,[29] and used the cclib library for post-analysis.[30]

As a measure for the reaction rates of any of the putative 48 reactions (3 reactants azide/Me-Tet/H-Tet times eight different enantiomers and diastereomers of TCO*/SCO/BCN, times two different relative tail orientations, Table S1), we calculated the activation energy *E*_act_ along the reaction pathway (Figure S2). Conventionally, and also as previously reported for this class of reactions,[19] *E*_act_ is defined as the difference between the transition state (TS) energy and the sum of the energies of geometry optimized reactants, *E*_act,Σreact_ = *E*_TS_−Σ*E*_react,opt_. Here, we instead use the energy of the van der Waals complex formed by the two reactants, *E*_vdW_, as a reference, that is, defined *E*_act_=*E*_act,vdW_=*E*_TS_ − *E*_vdW_ (Figure S2), the reason for which was two-fold. First, TS conformations and van der Waals complexes are affected by basis set superposition errors (BSSE), whereas the conformations of the smaller single reactants are less affected. Thus, the BSSE is inherently included in *E*_act,Σreact_ but largely cancels out in *E*_act,vdW_. In addition, we found that Σ*E*_react,opt_ is larger than *E*_TS_ for some of the fastest reactions involving TCO*, which would imply the absence of any activation barrier and is an artefact from overlooking the formation of a favourable van der Waals complex prior to the reaction. Nevertheless, we found a correlation of *E*_act,vdW_ with *E*_act,Σreact_ (Figure S4), and report *E*_act,vdW_ as activation energies in the following. Orbital energies were computed using the HF/6-311+G(d,p) level of theory and we define Δ*E*_FMO_ as the energy difference between two relevant interacting orbitals.

For computational efficiency, smaller compounds compared to the experimental reactants[9, 25] were used, but including a significant fraction of the handles and therefore still more detailed when compared to the highly simplified scaffolds of previous computations. The tetrazines R-group features a benzylamine, the azide a N-propyl, and the eight-ring an *N*-methylcarbamate group.

While for the DFT calculations we considered all conformers separately, experimental rates were obtained with mixtures of the TCO*a, TCO*e and SCO enantiomers, using stopped-flow spectroscopy (see Supporting Information). This resulted in a total of 15 measured reactions rates directly comparable to with our calculated barriers (see Supplemental Material for details).

## Results and Discussion

The understanding of click-chemistry reactions at their highest level of detail is crucial to improve their applicability in biomolecule labeling at the base of single-molecule spectroscopy. Therefore, azide/tetrazine cycloadditions to cyclooctenes and cyclooctynes were herein investigated taking into account the effect of R-groups used for proteins functionalization.

We obtained M06-2X energies from optimization of all stereoisomers including enantiomeric pairs of the reactants’ set (Figure S1). These energies suggest TCO*e to be more stable than TCO*a by 1.1 kcal mol^−1^, which also is the less reactive molecule among the tetrazine cycloadditions (Figure 1 a), but TCO*e undergoes more readily *cis*-isomerisation.[25]

We next calculated conformations and energies of van der Waals complexes and transition states for all possible reactions to estimate energy barriers *E*_act_. We obtained a high correlation between measured rates and calculated energy barriers (Figure 1 b, *R*^2^=0.89), validating our quantum mechanical calculations. Due to the sterically demanding protein that the eight-membered rings are attached to, the azide and tetrazine substituents are prone to orient in an antiparallel fashion to the carbamate sidechain of the 8-ring. Thus the energy barriers of such antiparallel oriented linker configurations were chosen here. Although a similar correlation between experimental rates and calculated activation energies was obtained when the side-chain regioselectivity was ignored (Figure S3), individual barriers can vary by up to 3 kcal mol^−1^ when changing tail orientation (Table S1). This suggests the steric demand of the linkers including the bulky label and biomolecule to crucially determine the reaction kinetics.

**Figure 2:**
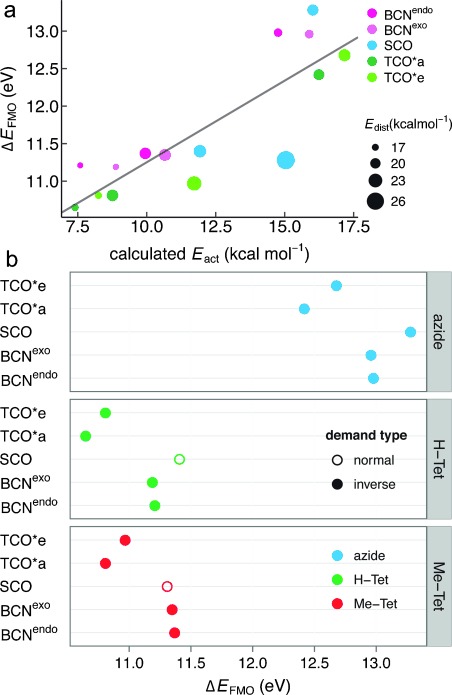
Electron demand determines cycloaddition rate. a) Correlation between FMO energy differences (Δ*E*_FMO_) of the 15 distinct reactions (*E*act is the barrier for the two enantiomers obtained in antiparallel tail orientation). The dot size represents the summed distortion energies of both reactants. The solid line shows the linear fit of the data. b) Electron demand of the 15 different reactant pairs according to FMO energy gaps.

To identify the origin of the differences in reactivity, we analyzed the energy differences Δ*E*_FMO_ between interacting frontier molecular orbitals (FMO) of all 15 reactant pairs (for details see Supporting Information). The relevant FMOs and energies are listed in Tables S2 and S3. Overall Δ*E*_FMO_ correlates very well with *E*_act_ (*R*^2=^0.72, Figure 2 a). As expected, Δ*E*_FMO_ systematically underestimates the barrier for ligations with high distortion energies (dot size in Figure 2 a). We obtained an improved correlation when comparing the sum of distortion energies and Δ*E*_FMO_ with *E*_act_ (*R*^2^=0.82), implying that in a first approximation these two contributions can be considered additive and are both critically determining reactivitiy.

**Figure 3:**
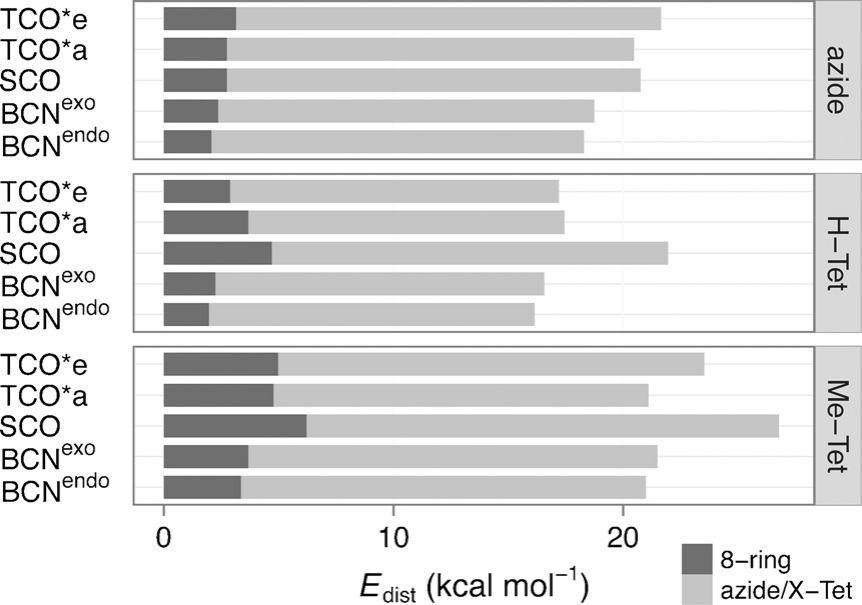
Distortion energies *E*_dist_ from the vdW complex to the transition state conformation, decomposed for the ring and azide/tetrazine compounds (see Methods for details).

The main contribution to distortion energies generally comes from the azide or tetrazine, respectively (Figure 3). Tetrazine (Me-Tet and H-Tet) ligations to SCO as well as any of the cycloadditions involving Me-Tet show significantly higher distortions than the other cycloadditions (Figures 2 a and 3), suggesting that the methyl group of Me-Tet as well as the carbamate right next to the triple bond of SCO creates a steric hindrance for the transition state formation.3

We also analyzed the origins for the different observed reactivity between the investigated reactions. As expected, Δ*E*_FMO_ are overall higher in cycloadditions involving azides rather than tetrazines, explaining the well-known fact that *n*-propyl azide reacts less efficiently overall (Figure 2 a).[13–14] Our calculations and measurements also confirm the previously established higher preference of this azide with BCN^*endo*/*exo*^ over SCO,[2, 31] for which we predict a ∼3 kcal mol^−1^ difference in barrier. Our results further agree with the higher reactivity of azide with BCN^*endo*^ over BCN^*exo*^,[32] which we find to similarly hold for H-Tet and Me-Tet (Figure 2 a).

We can now ascribe the smaller barrier for the cycloaddition of the azide with BCN to a smaller HOMO–LUMO gap between the two reactants (Figure 2 b). According to both calculations and measurements, TCO*a reacts faster than TCO*e with both H-Tet (∼3-fold) and Me-Tet (∼700-fold, Figure 1 b), again directly in line with the smaller FMO energy gap for the TCO*a isomer (Figure 2 b). The axial position increases the electron-withdrawing effect of the carbamate group, an effect that is further enhanced by the smaller distortion required for the ligation of TCO*a to a tetrazine (Figure 3).

**Figure 4:**
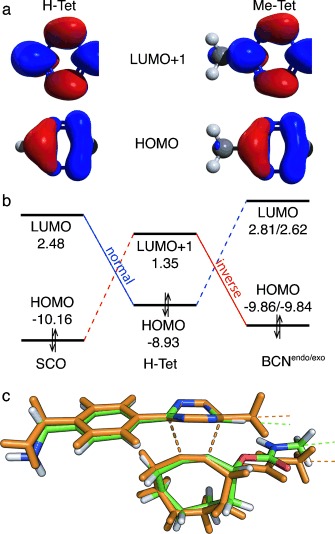
Origin of differences in electron demand and distortion a) The methyl group of Me-Tet shifts electron density into the tetrazine ring, as evidenced by the differences in shape of the LUMO and HOMO between H-Tet and Me-Tet (for clarity only the substituted tetrazine ring is shown). b) Energy gaps between FMOs of H-Tet reacting with SCO and BCN^*endo*/*exo*^. SCO features lower FMO energies than BCN^*endo*/*exo*^ and undergoes cycloadditions with H- Tet (and Me-Tet, see Table S3) with normal electron demand (solid blue line) instead of inverse electron demand as for BCN^*endo*/*exo*^ (solid red line). The energy levels are not drawn to scale. c) The transition state of SCO-Me-Tet (orange) shows a significantly higher distortion than the one of SCO-H-Tet (green, compare orange and green dashed lines) resulting in a larger asymmetry with regard to the forming of C–C bonds (darshed yellow lines).

Reactions involving H-Tet are generally faster than those with Me-Tet, a trend to be expected in this case of inverse electron demand of SPIEDAC reactions, as the methyl group shifts electron density into the reacting 6-ring (Figure 4 a). Surprisingly, the so-called SPIEDAC involving SCO and a tetrazine[9] we instead predict to proceed with normal electron demand and correspondingly term this reaction SPINEDAC. The carbamate group makes SCO more electrophilic than BCN, rendering the interaction of its LUMO with the tetrazine HOMO more favorable (Figure 4 b). SCO ligation to Me-Tet nevertheless is slower than to H-Tet, because the sterically more demanding methyl group gives rise to a 6 kcal mol^−1^ higher distortion of the transition state (Figures 2 and 4 c). We therefore propose that this SPINEDAC reaction can be sped up conversely to SPIEDAC reactions, namely by more strongly electron-drawing cyclooctyne substituents and/or by further electron-donating tetrazine substituents, both with as little steric demand as possible. Our results, however, also emphasize that care must be taken as reactions can switch between inverse and normal electron demand upon allegedly minuscule chemical changes.4

## Conclusion

We here analyzed the determinants of reactivity for a set of strain-promoted cycloaddition reaction partners that is identical to those used for in vitro and in vivo labeling.

We could show that handles not only play the role to attach the reactive moieties to fluorescent labels and proteins. Instead, they also decisively influence the reaction rate of the cycloadditions, due to their electronic properties and steric demand. Our data explain how different isomeric states, methyl groups and carbamate linkers can independently and decisively alter the speed of SPAAC, SPIEDAC and the newly defined SPINEDAC reactions. Most importantly, the presence of the methyl and/or benzyl moieties of H-Tet and Me-Tet together with the electron-withdrawing properties of the carbamate handle of the SCO cyclooctyne can switch the electron demand from an inverse to a normal type, with direct consequences for the rational tuning of the reactivity of these reactions.

A more strongly electron-donating group replacing the methyl or benzyl groups of H-Tet/Me-Tet will increase the reactivity of ligations with SCO, but not with TCO* and BCN. Even modifications of the aminogroup at the benzyl ring of H-Tet/Me-Tet, although distant, can modulate the electron density of the benzene and thereby the reactivity of the tetrazines. Likewise, replacing the carbamate handle with more strongly electron-withdrawing groups, is predicted to have a similar effect. Benzyl and methyl moieties at Me-Tet and H-Tet are sterically highly demanding. Their substitution by smaller and/or electron-withdrawing groups should considerably enhance SPIEDAC, but not SPINEDAC reactions. These structural features should thus be taken into account when re-engineering cycloadditions in computations and experiments.

## Supporting Information

As a service to our authors and readers, this journal provides supporting information supplied by the authors. Such materials are peer reviewed and may be re-organized for online delivery, but are not copy-edited or typeset. Technical support issues arising from supporting information (other than missing files) should be addressed to the authors.

miscellaneous_information
