# Supplementary material for: Origin of Orthogonality of Strain-Promoted Click Reactions
Source: Chemistry. 2015 Jul 14;21(35):12431–5. doi: 10.1002/chem.201501727 (PMC4600239; doi:10.1002/chem.201501727)
Supplement: Supplementary file 1 — miscellaneous_information [file chem0021-12431-sd1.pdf]

CHEMISTRY
A European Journal

Supporting Information

Origin of Orthogonality of Strain-Promoted Click Reactions

Johannes A. Wagner,[a, b] Davide Mercadante,[a, c] Ivana Nikić,[d] Edward A. Lemke,[d] and Frauke Gräter*[a, c]

chem_201501727_sm_miscellaneous_information.pdf

Supplementary Methods

DFT calculations

Activation barriers were calculated for reactions between the following compounds (Suppl. Figure 1), named here according to IUPAC nomenclature: n-propyl azide,
(4-(1,2,4,5)-tetrazin-3-yl)phenyl methanamine or benzylamino tetrazine (H-Tet) and
(4-(1,2,4,5)-6-methyl-tetrazin-3-yl)phenyl methanamine or benzylamino methyltetrazine (Me-Tet) with
R,2E,R_p- and S,2E,S_p-cyclooct-2-en-1-methylcarbamate (TCO*e),
R,2E,S_p- and S,2E,R_p-cyclooct-2-en-1-methylcarbamate (TCO*a),
R- and S-cyclooct-2-yn-1-methylcarbamate (SCO), and
endo- and exo-bicyclonon-5-yn-1-methylcarbamate (BCN^{endo} & BCN^{exo}).

Initial conformations of all 11 reactants (Figure 1a, Suppl. Figure 1) were obtained by steepest descent and conjugate gradient energy minimization starting from a large rotamer ensemble of each reactant using the general amber force field (GAFF),[1,2] deploying the software Avogadro.[3] The lowest energy conformations were then used as input coordinates for quantum mechanical (QM) optimization using the M06-2X Minnesota functional[1] and the Pople split valence basis set 6-311+G(d,p)[4] deploying Gaussian 09.[5] This Hybrid-Meta exchange correlation functional features medium range dispersion interaction and is known to perform well for these type of reactions at moderate computational cost.[6–9] The M06-2X functional is known to underestimate dispersion interactions such as those of benzene in Me-Tet and H-Tet.[10,11] However, this error should largely cancel out in our calculations as the van der Waals complex that we chose as the reference state for E_{act} has dispersion interactions highly similar to the one of the TS. We performed calculations in both vacuum and solvent, and report the experimentally more relevant results from calculations in solvent only. For several reactions, very different conformations were obtained in solvent calculations vs. vacuum, hence computing solvated energies on conformations obtained from vaccum calculations was not feasible. QM data was parsed for further analysis using the excellent cclib library.[12] Upon ligation of each of the eight 8-membered ring isomers to azide, Me-Tet and H-Tet, respectively, in both an antiparallel and parallel fashion with respect to the tail orientations of the two reactants, we obtained 48 products, which were again minimized using M06-2X.

Due to the multi-configurational phase space of the complex molecules investigated here, obtaining global minima and real TS structures poses a challenge. The transition state (TS) search for each of the reactions was performed through relaxed potential energy surface (PES) scans in reverse reaction coordinate direction, at the full M06-2X/6-311+G(d,p) level of theory. For SPAAC reactions, one-dimensional PES scans were performed by opening the N-N-N azide angle in steps of 2 degrees, refined to 0.5 degrees close to the saddle point. The two highest energy conformations from the PES scans were then used as starting points for a TS search employing the Berny algorithm. SPIEDAC/SPINEDAC scans were performed along two dimensions by increasing the lengths of the two newly formed C-N bonds by about 0.2 Å per step. Here, three scan points in the vicinity of the saddle point were used as starting points for the TS search. While not always all three starting points lead to a TS structure as indicated by a single imaginary frequency, with SPIEDAC/SPINEDAC reactions being particularly challenging, we could finally obtain TS for all 48 reactions from at least

two different starting points in the scanned PES. The imaginary frequencies of the transition states are listed in Suppl. Table 1.

Van der Waals complexes of all reactions were obtained by geometry optimizing a conformation close to the TS with the PES scan coordinates slightly above the TS treshold. All of the obtained geometry optimized conformations (reactants, complexes, and products) and their energies were validated by additional optimizations starting from other initial configurations.

The ligation of Me-Tet and H-Tet to the strained rings is followed by a low barrier N_2 exit (for which we estimate a height of 1.5-2 kcal/mol), so that the first cycloaddition step represents the rate-limiting step. This is in agreement with previous studies.[13]

We identified the set of possible interacting molecular orbitals by symmetry overlaps between the relevant occupied and unoccupied orbitals for any pair of reactants. The smallest energy difference defined the interacting orbitals and thereby if a normal (diene HOMO with dienophile LUMO) or inverse demand Diels-Alder reaction (diene LUMO with dienophile HOMO) is at play (Suppl. Tables 2 and 3). Figures of interacting orbitals are provided for download as additional Supplemental Material.

The kinetic measurements were performed using stopped-flow spectroscopy. Details are described elsewhere,[14] and results are listed in Suppl. Table 1.

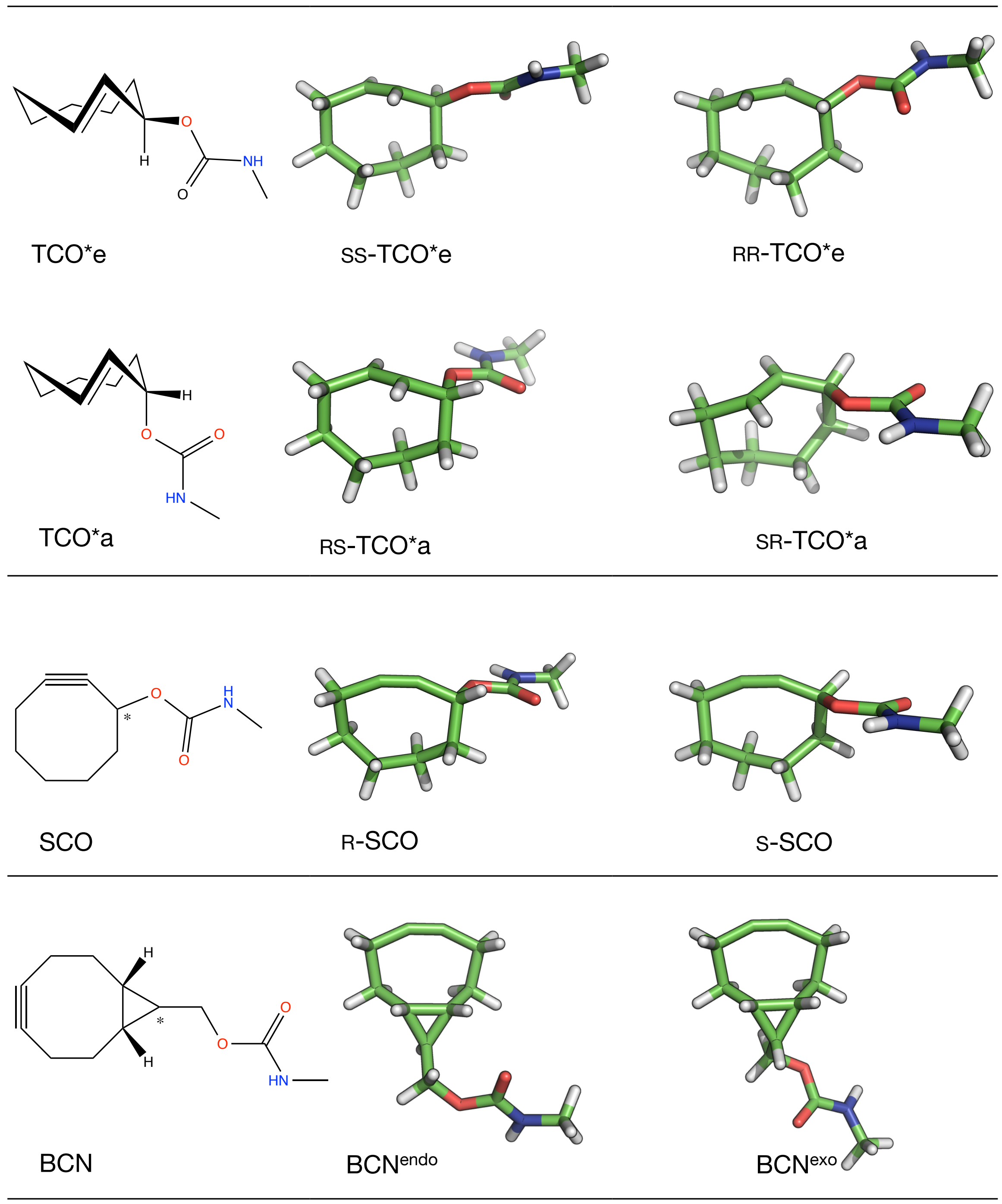

Supplemental Figure 1: Structures and naming scheme of M06-2X/6-311+G(d,p) optimized conformations of all 8-member ring confomers.

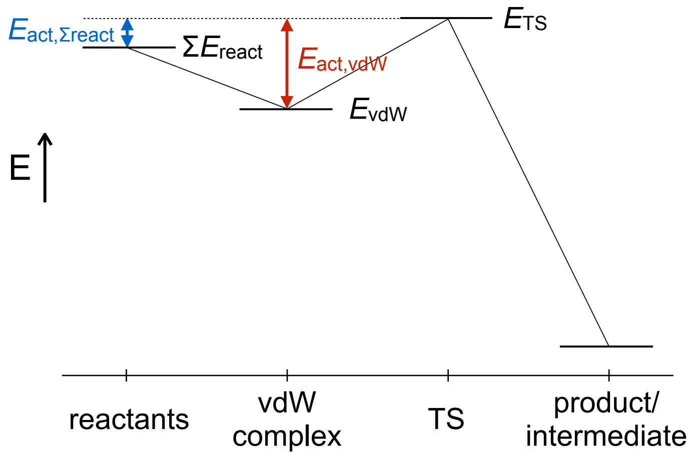

Supplemental Figure 2: Reaction pathway. E_{act} was calculated relative to the van der Waals complex' energy as opposed to the sum of the isolated reactants energies.

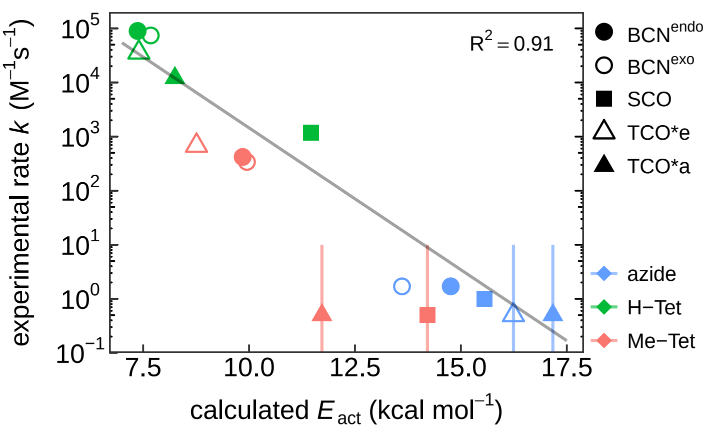

Supplemental Figure 3: Correlation between experimental reaction rates k and the calculated activation energies E_{act} for each pair of reactants. In contrast to Figure 1b of the main text (only antiparallel tail orientations), we here chose the lower E_{act} among antiparallel and parallel tail orientations, ignoring regioselectivity. Even in this case we obtain an excellent correlation of 0.91 with the experimental rates. Only the lowest E_{act} among the different enantiomers (of SCO and TCO*) are shown. Experimental error bars are smaller than the symbol size and are omitted for clarity.

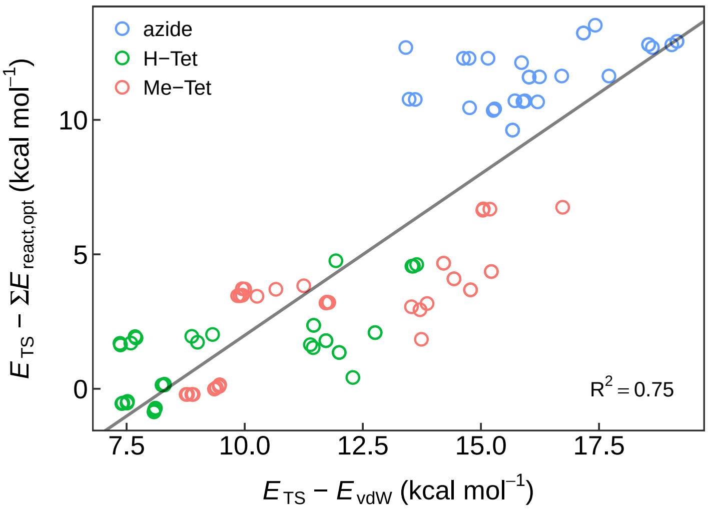

Supplemental Figure 4: Correlation of vdW complex activation energies with those using the sum of reactant energies $\sum E_{\text{react,opt}}$ as a reference. The line depicts the linear regression over all energies and has a slope of 1.2.

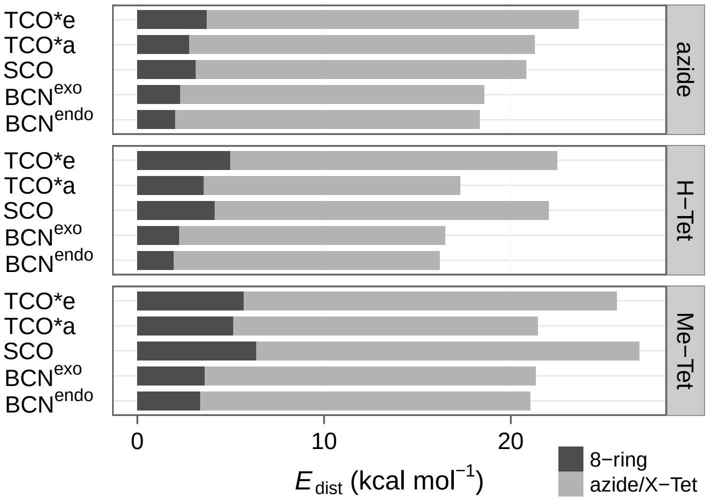

Supplemental Figure 5: Decomposed distortion energies for all reactions analogous to Figure 3 in the main text, but for regioselectivity in parallel tail orientation. Particularly, all equatorial TCO's (TCO*e) show a higher distortion energy compared to the antiparallel tail orientation.

reactant 1	reactant 2	E_{act} (kcal·mol^{-1})	ΔE_{FMO} (eV)	measured k (M^{-1}·s^{-1})	error k (M^{-1}·s^{-1})	$E_{dist,ring}$ (kcal·mol^{-1})	$E_{dist,azide/tet}$ (kcal·mol^{-1})	$\sum E_{dist}$ (kcal·mol^{-1})	Img freq (cm^{-1})
regioselectivity: antiparallel tail orientation									
azide	TCO*e	17.17	12.68	n.d.	n.d.	3.15	18.49	21.64	-457.3
azide	TCO*a	16.24	12.42	n.d.	n.d.	2.75	17.72	20.47	-457.0
azide	SCO	16.02	13.28	1.0	-	2.75	18.01	20.76	-454.7
azide	BCN^{endo}	14.76	12.98	1.7	0.3	2.08	16.21	18.29	-434.6
azide	BCN^{exo}	15.89	12.96	1.7	0.3	2.37	16.37	18.74	-436.9
H-Tet	TCO*e	8.25	10.81	12080	150	2.89	14.31	17.20	-311.6
H-Tet	TCO*a	7.40	10.65	35900	420	3.68	13.76	17.44	-289.9
H-Tet	SCO	11.93	11.41	1180	80	4.71	17.24	21.95	-372.7
H-Tet	BCN^{endo}	7.50	11.21	89700	3200	1.97	14.17	16.14	-297.0
H-Tet	BCN^{exo}	7.68	11.19	74090	1100	2.25	14.31	16.56	-299.8
Me-Tet	TCO*e	11.72	10.97	n.d.	n.d.	4.98	18.55	23.53	-350.7
Me-Tet	TCO*a	8.76	10.81	680	5	4.78	16.32	21.10	-326.6
Me-Tet	SCO	15.04	11.31	n.d.	n.d.	6.22	20.56	26.78	-394.4
Me-Tet	BCN^{endo}	9.95	11.37	420	10	3.36	17.63	20.99	-335.2
Me-Tet	BCN^{exo}	10.66	11.35	340	10	3.68	17.81	21.49	-337.9
regioselectivity: parallel tail orientation									
azide	TCO*e	19.04	12.68			3.72	19.91	23.63	-471.5
azide	TCO*a	18.55	12.42			2.78	18.51	21.29	-459.7
azide	SCO	15.56	13.28			3.13	17.70	20.83	-443.2
azide	BCN^{endo}	15.29	12.98			2.04	16.30	18.34	-437.0
azide	BCN^{exo}	13.61	12.96			2.31	16.27	18.58	-434.9
H-Tet	TCO*e	11.39	10.81			4.99	17.50	22.49	-347.9
H-Tet	TCO*a	8.08	10.65			3.57	13.72	17.29	-288.2
H-Tet	SCO	11.46	11.41			4.14	17.89	22.03	-375.1
H-Tet	BCN^{endo}	7.37	11.21			1.95	14.23	16.18	-295.7
H-Tet	BCN^{exo}	7.68	11.19			2.24	14.25	16.49	-296.3
Me-Tet	TCO*e	13.53	10.97			5.71	19.98	25.69	-369.9
Me-Tet	TCO*a	9.40	10.81			5.15	16.30	21.45	-325.8
Me-Tet	SCO	14.21	11.31			6.37	20.50	26.87	-389.3
Me-Tet	BCN^{endo}	9.85	11.37			3.38	17.66	21.04	-336.7
Me-Tet	BCN^{exo}	9.95	11.35			3.62	17.71	21.33	-336.3

Supplemental Table 1: Overview of all activation energies, orbital gaps, distortion energies for the 8-ring and the azide or tetrazine, imaginary frequencies of the TS for all isomers and tail orientations and measured experimental rates k. The measured reaction rates are for H-Tet-Cy5 and Me-Tet-Cy5, respectively (see[14] for more details). The reaction rates for the azide-SCO/BCN cycloadditions were taken from Borrmann et al..[15]

	MO and corresponding energies in (eV)			
compound	HOMO-1	HOMO	LUMO	LUMO+1
azide	-12.021	-10.260	3.116	4.063
H-Tet	-11.153	-8.929	0.765	1.346
Me-Tet	-10.989	-8.821	0.823	1.508
TCO*e	-11.807	-9.457	2.859	3.620
TCO*a	-11.812	-9.301	2.757	3.710
SCO	-10.550	-10.158	2.484	3.557
BCN^{endo}	-9.975	-9.859	2.805	3.317
BCN^{exo}	-9.966	-9.839	2.616	2.754

Supplemental Table 2: FMO energies in (eV) computed at HF/6-311+G(d,p) level of theory on geometry optimized structures.

reactant 1	orbital 1	reactant 2	orbital 2	ΔE_{FMO} (eV)
azide	LUMO	TCO*e	HOMO	12.68
azide	LUMO	TCO*a	HOMO	12.42
azide	LUMO	SCO	HOMO	13.28
azide	LUMO	$\mathrm{BCN}^{\mathrm{endo}}$	HOMO	12.98
azide	LUMO	$\mathrm{BCN}^{\mathrm{exo}}$	HOMO	12.96
H-Tet	LUMO+1	TCO*e	HOMO	10.81
H-Tet	LUMO+1	TCO*a	HOMO	10.65
H-Tet	HOMO	SCO	LUMO	11.41
H-Tet	LUMO+1	$\mathrm{BCN}^{\mathrm{exo}}$	HOMO	11.21
H-Tet	LUMO+1	$\mathrm{BCN}^{\mathrm{exo}}$	HOMO	11.19
Me-Tet	LUMO+1	TCO*e	HOMO	10.97
Me-Tet	LUMO+1	TCO*a	HOMO	10.81
Me-Tet	HOMO	SCO	LUMO	11.31
Me-Tet	LUMO+1	$\mathrm{BCN}^{\mathrm{endo}}$	HOMO	11.37
Me-Tet	LUMO+1	$\mathrm{BCN}^{\mathrm{exo}}$	HOMO	11.35

Supplemental Table 3: ΔE_{FMO} of interacting orbitals in eV. The red rows indicate normal demand Diels-Alder reactions, all others are inverse electron demand.

References

[1] J. Wang, R. M. Wolf, J. W. Caldwell, P. A. Kollman, D. A. Case, *J Comput Chem* **Apr. 2004**, *25*, 1157–1174.

[2] J. Wang, W. Wang, P. A. Kollman, D. A. Case, *J Mol Graph Model* **Oct. 2006**, *25*, 247–260.

[3] M. D. Hanwell, D. E. Curtis, D. C. Lonie, T. Vandermeersch, E. Zurek, G. R. Hutchison, *J Cheminform* **2012**, *4*, 17.

[4] R Krishnan, J. S. Binkley, R Seeger, J. A. Pople, *J Chem Phys* **1980**, *72*, 650.

[5] M. J. Frisch, G. W. Trucks, H. B. Schlegel, G. E. Scuseria, M. A. Robb, J. R. Cheeseman, G. Scalmani, V. Barone, B. Mennucci, G. A. Petersson, H. Nakatsuji, M. Caricato, X. Li, H. P. Hratchian, A. F. Izmaylov, J. Bloino, G. Zheng, J. L. Sonnenberg, M. Hada, M. Ehara, K. Toyota, R. Fukuda, J. Hasegawa, M. Ishida, T. Nakajima, Y. Honda, O. Kitao, H. Nakai, T. Vreven, J. A. Montgomery, Jr., J. E. Peralta, F. Ogliaro, M. Bearpark, J. J. Heyd, E. Brothers, K. N. Kudin, V. N. Staroverov, R. Kobayashi, J. Normand, K. Raghavachari, A. Rendell, J. C. Burant, S. S. Iyengar, J. Tomasi, M. Cossi, N. Rega, J. M. Millam, M. Klene, J. E. Knox, J. B. Cross, V. Bakken, C. Adamo, J. Jaramillo, R. Gomperts, R. E. Stratmann, O. Yazyev, A. J. Austin, R. Cammi, C. Pomelli, J. W. Ochterski, R. L. Martin, K. Morokuma, V. G. Zakrzewski, G. A. Voth, P. Salvador, J. J. Dannenberg, S. Dapprich, A. D. Daniels, O. Farkas, J. B. Foresman, J. V. Ortiz, J. Cioslowski, D. J. Fox, Gaussian 09 Revision B.01, Gaussian Inc. Wallingford CT 2009.

[6] E. G. Hohenstein, S. T. Chill, C. D. Sherrill, *J. Chem. Theory Comput.* **Dec. 2008**, *4*, 1996–2000.

[7] L. Goerigk, S. Grimme, *Phys. Chem. Chem. Phys.* **2011**, *13*, 6670.

[8] Y. Liang, J. L. Mackey, S. A. Lopez, F. Liu, K. N. Houk, *J. Am. Chem. Soc.* **Oct. 2012**, *134*, 17904–17907.

[9] Y. Lan, L. Zou, Y. Cao, K. N. Houk, *J. Phys. Chem. A* **Dec. 2011**, *115*, 13906–13920.

[10] N. Marom, A. Tkatchenko, M. Rossi, V. V. Gobre, O. Hod, M. Scheffler, L. Kronik, *J. Chem. Theory Comput.* **Dec. 2011**, *7*, 3944–3951.

[11] R. Sedlak, T. Janowski, M. Pitonak, J. Rezac, P. Pulay, P. Hobza, *J. Chem. Theory Comput.* **Aug. 2013**, *9*, 3364–3374.

[12] N. M. O'boyle, A. L. Tenderholt, K. M. Langner, *J Comput Chem* **2008**, *29*, 839–845.

[13] W. Chen, D. Wang, C. Dai, D. Hamelberg, B. Wang, *Chemical Communications* **2012**, *48*, 1736–1738.

[14] J.-E. Hoffmann, T. Plass, I. Nikic, I. Valle Aramburu, C. Koehler, H. Gillandt, E. A. Lemke, C. Schultz, *Chem. Eur. J.* **2015**, submitted.

[15] A. Borrmann, S. Milles, T. Plass, J. Dommerholt, J. M. M. Verkade, M. Wiessler, C. Schultz, J. C. M. van Hest, F. L. van Delft, E. A. Lemke, *ChemBioChem* **Sept. 2012**, *13*, 2094–2099.
